# DoE Approach to Setting Input Parameters for Digital 3D Printing of Concrete for Coarse Aggregates up to 8 mm

**DOI:** 10.3390/ma16093418

**Published:** 2023-04-27

**Authors:** Arnošt Vespalec, Jan Podroužek, Daniel Koutný

**Affiliations:** 1 Faculty of Mechanical Engineering, Institute of Machine and Industrial Design, Brno University of Technology, Technická 2896/2, 616 69 Brno, Czech Republic; arnost.vespalec@vut.cz (A.V.);; 2 Faculty of Civil Engineering, Institute of Computer Aided Engineering and Computer Science, Brno University of Technology, Veveří 331/95, 602 00 Brno, Czech Republic

**Keywords:** 3DCP, inverse material characterisation, large-scale additive manufacturing, contour crafting, digital manufacturing, cementitious material, coarse aggregate concrete printing

## Abstract

This paper is primarily concerned with determining and assessing the properties of a cement-based composite material containing large particles of aggregate in digital manufacturing. The motivation is that mixtures with larger aggregate sizes offer benefits such as increased resistance to cracking, savings in other material components (such as Portland cement), and ultimately cost savings. Consequently, in the context of 3D Construction/Concrete Print technology (3DCP), these materials are environmentally friendly, unlike the fine-grained mixtures previously utilized. Prior to printing, these limits must be established within the virtual environment’s process parameters in order to reduce the amount of waste produced. This study extends the existing research in the field of large-scale 3DCP by employing coarse aggregate (crushed coarse river stone) with a maximum particle size of 8 mm. The research focuses on inverse material characterization, with the primary goal of determining the optimal combination of three monitored process parameters—print speed, extrusion height, and extrusion width—that will maximize buildability. Design Of Experiment was used to cover all possible variations and reduce the number of required simulations. In particular, the Box—Behnken method was used for three factors and a central point. As a result, thirteen combinations of process parameters covering the area of interest were determined. Thirteen numerical simulations were conducted using the Abaqus software, and the outcomes were discussed.

## 1. Introduction

Today, the construction industry is expanding at an alarming rate, resulting in the overexploitation of limited natural resources and the massive emission of greenhouse gases [1]. According to the International Energy Agency (IEA), 39% of global CO_2_ emissions in 2019 come from the construction industry. Unfortunately, the production of these greenhouse gases is on the rise [2]. Thus, in the near future, we will inevitably face climate change, which already poses a global threat to the environment. As a result of these changes, there will be a greater emphasis on reducing greenhouse gas emissions and sustainable economic development linked to a circular economy and the efficient use of building materials. Since the 1990s, there has been an exponential increase in the number of research and development projects aimed at finding solutions for sustainable building production and design. As a result, it is only logical that the private and academic sectors of the construction industry will respond [3]. Using the capability to 3D print freeform and complex structures that are virtually impossible to produce using conventional methods, the construction industry will undergo a radical transformation in the near future [4,5]. Ultimately, 3D concrete printing (3DCP) technology can lead to a more sustainable building, primarily due to the potential reduction in construction material consumption compared to conventional methods [6]. However, material savings using 3DCP technology is a multi-level problem in which a balance must be struck between material composition, material consumption, and the appropriate level of complexity of the printed geometry. This is ultimately influenced by 3D printing methods. To accomplish sustainable development, it is necessary to evaluate the impact of our actions on both the environment and human health. This involves analysing each stage of the production chain, including the manufacturing, distribution, and use of both conventional and 3D printing construction and architecture technologies, while considering both direct and indirect effects. Every aspect of the process must be considered, from mining natural resources to refining fundamental raw materials such as cement, sand, aggregates, and additives, to construction design and utilization, maintenance, duration, and recycling. In addition, we must evaluate the distribution of traffic, the management of refuse, and the application of advanced design tools such as structural optimization and shape complexity with functional hybridization. By incorporating 3D printing technology into the equation, we can generate new iterations of structures that take all these factors into account, resulting in material savings. Through this comprehensive strategy, we can aspire for a future that is more sustainable for everyone, not just businesses [6,7]. In architecture and construction, 3DCP technology utilizes cement-based mixtures in the form of mortar. They consist solely of fine aggregate with a maximum size of four millimetres, organic admixtures and predominantly plastic fibre reinforcement [8,9,10,11,12]. These mortars are not environmentally sustainable in terms of additive manufacturing on a large scale, in terms of saving global resources, and ultimately reducing costs [13]. Currently, we can observe scientific endeavours that are beginning to investigate a mixture consisting of up to 8mm-sized large aggregates. Mentionable are Ice Industries [14], Brno University of Technology [7,15], and the Danish Technological Institute [16], which have emphasized the ongoing development and research of printing with a mixture containing a large aggregate fraction.

Currently, there is a clear trend towards printing with non-Portland cement binders, which significantly reduce CO_2_ emissions (approximately 80%) and energy consumption (approximately 60%) [17,18,19,20]. Although these advanced materials point the way to the future, substantial materials science research is still required to bring them to the same level of stability as cement-based materials for real-world applications [8,21]. Despite the consumption of cement concrete and the significant carbon footprint of its production, which has a negative effect on the environment [22,23], its use is generally justified. Nevertheless, even with current 3DCP methods, a significant quantity of waste is generated, reducing the technology’s efficiency, and increasing its carbon footprint [24,25]. By setting the input parameters appropriately, the entire process can be managed more efficiently, and the waste associated with 3D printing can be reduced [26]. 

In this paper, a set of factors that influence the success of the 3DCP process will be defined and these constraints will be quantified for a mixture containing coarse aggregates with maximum size of 8 mm. The objective is to identify the entire design space and determine the optimal solution using the numerical simulation tools Cobra-Print plugin and Abaqus software [27], thereby avoiding the common trial-and-error approach in 3DCP technology. It is an actual topic, so we can observe scientific teams investigating the use of numerical simulations for the buildability quantification of 3DCP models that roughly approximate reality [28,29]. DoE principles will be implemented in order to determine the entire hypothetically infinite boundary of the design space, reduce the number of simulations, and ultimately reduce computational requirements, costs, and human resources. The results will permit input parameters to be analyzed prior to actual implementation, thereby decreasing the number of failed implementations.

## 2. Literature Review—Theoretical Framework

The main quality criterion for 3DCP is the buildability, which is dependent on two failure modes that have been identified. They are failure of a material by plastic yielding and failure by elastic buckling due to local or global instability [30,31]. The evolution of the order of these two modes depends on controllable (print object geometry—design, printing strategy) and uncontrollable (time-dependent material evolution) factors. Fresh printable material exhibits a thixotropic behavior that varies with time even in dormant periods, according to global research. In addition, research involving cementitious binders revealed that the modulus of strength and elasticity increases linearly with time. Optimally there should be no elastic and plastic deformations during printing. Nevertheless, such ideal conditions cannot be realized. Priority should be placed on ensuring that the process parameters are optimally set to minimize the occurrence of these deformations during printing. As stated previously, buildability is dependent on a variety of parameters and their appropriate settings, which can make the entire process significantly more efficient. Based on the literature, three factors that influence the 3DCP process were identified and will be discussed below: technical equipment and material selection (Section 2.1), the setting of printing parameters (Section 2.2), the simulation prior to printing (Section 2.3), and the novelty contribution of this study (Section 2.4). 

### 2.1. Technical Equipment and Material Selection

**Figure 1:**
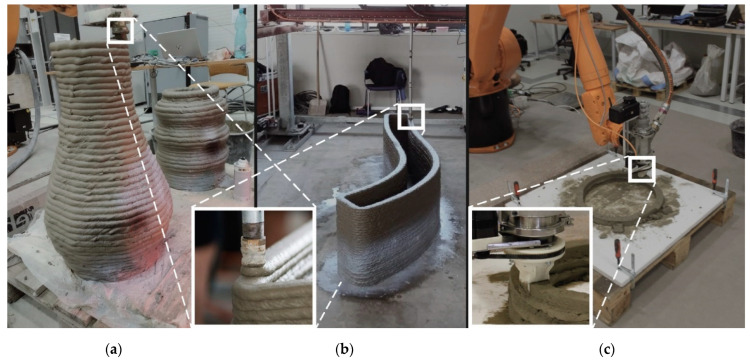
Technical equipment: Robotic arm KUKA and gantry large format 3D printer with extrusion method of print (**a**,**b**), and (**c**) robotic arm KUKA with CC method of print.

In the beginning, Additive Manufacturing (AM) technology utilized gantry printers, and this printer type is still used in some applications [8]. The primary benefit of gantry printers is printing accuracy, which is not essential for the majority of construction projects. Due to the limited size of the printed object when using gantry printers, more sophisticated techniques for 3DCP have been developed. This type of printer typically operates with three or four degrees of freedom, therefore the full potential of 3DCP technology is not realized (see Figure 1). Following this, robotic arms and the cable driven robot were created. The main advantage of the robotic arm is its ability to print more complex shapes due to its six axes (Figure 1a). Some projects required unusual printing applications, for example on the ceiling. The disadvantage of robotic arms is generally a lower load capacity and accuracy compared to gantry printers, but for the construction industry the accuracy is sufficient.

A cable-driven robot can print very large objects [32]. In terms of degrees of freedom, this printer type is intermediate between a gantry printer, and a robotic arm. The main issue with cable-driven robots is the printing speed limitation associated with precision, which is frequently exacerbated by the springy cables. Printing accuracy is often compensated for by active software correction. The operation of a cable-driven parallel robot is therefore difficult in terms of preparing and programming [33]. 

In addition, the method of extrusion affects printing, and different methods may be more suitable for different materials. The diameter of the extrusion outlet should correspond to the largest aggregate in the mixture. The cross-section of the extruder nozzle is typically recommended to be three to five times the maximum size of the aggregate. Contour Crafting (CC) (Figure 1c), D-shape, extrusion-based concrete printing (Figure 1a,b), and selective deposition of ultra-high-performance concrete [3] are the four most prevalent methods used for 3D printing technology in the architecture and construction industry. The CC printing method is optimal for preventing macroscopic imperfections that would lead to premature failure of the printed geometry and for enhancing the mechanical properties of the printed solid. According to the constant laminar flow of the extrusion maintained by the rectangular geometry of a nozzle and track shape supported by rectified trowels [18,34,35,36], it has a demonstrably greater shape stability of a printed track.

**Material Parameters**

Regarding the limitation of technological equipment in the laboratory and the requirements for the final product [7], it is necessary to select appropriate materials and at the same time prevent unnecessary trial-and-errors. This is intended to facilitate the use of numerical simulations, which have specific requirements for the description of the material in terms of describing its behavior with general expressions. Not all studies offer exhaustive generalizations of material properties required for numerical simulation. In general, a material’s behavior as a function of time, its hardening, and its cohesive properties must be described [27,30,31,37,38]. In addition, the literature review revealed that some studies [18,39,40,41] contained a great deal of information about the material but did not produce a specific material model. In addition, cement-based materials are most commonly used in 3DCP technology, due to the fact that cement-based materials have proven reliability based on long-term use.

A representative sample of studies that provide quite complete information for numerical simulation is recorded in Table 1, where the values of density, Poisson’s number, internal friction angle, dilation angle, and the time evolution equations for Young’s modulus and cohesion are given. These parameters are described in greater detail in Section 3.1. In some studies, printing was performed on multiple mixtures, then they are referred to as Mix 1, Mix 2, etc.

### 2.2. Setting of Printing Parameters

All of the studies listed in Table 2 were combed for information on print parameter configurations. Unfortunately, this information is not provided in the majority of instances. Some parameters were obtained only for the studies [16,27,30,37,38,40,42,43,44,45]. The working window t and print speed v are closely related; build speed (the time interval between successive layers) must be considered. Too slow a build rate causes the critical layer to reach stiffness and reduces the likelihood of plastic yielding, leading to low interlayer bonding [15,46], whereas too fast a build rate results in plastic failure, increased interlayer bonding, and poor surface quality [40]. This characteristic of layered concrete should be taken into account for large prints. The size of the print track must also be determined in relation to the object being printed. For greater stability, it is preferable to work with a wider and lower footprint; however, this is not economically advantageous, and the aspect ratio must be optimised. It is evident from the literature review that complete printing parameters are rarely reported in studies. In the majority of cases, the authors are concerned with printing speed, nozzle geometry and size, print track dimensions, etc., and there is still very little evidence regarding which parameters have the greatest impact on buildability with regard to a specific material.

**Printing Geometry**

A number of studies listed in Table 2 utilize different printing geometries, with the hollow cylindrical geometry being preferred for investigations of buildability. The hollow cylinder is composed of two right-circular cylinders that are connected to each other. It is hollow on the inside and the inner and outer radii are distinct. The axis point is perpendicular to the central base and is shared by both cylinders. This shape ensures a constant second moment of the surface about any axis, and provides continuous lateral self-support, thereby reducing the probability of the thin-walled structure tilting. In contrast, due to stress concentration at the corners, the square shape is unsuitable [40,47]. 

It should be noted that printing shape-complex geometries, reinforced walls [48], bio-inspired structures [49], etc., will additionally require a different approach to the strategy and methods used to generate print trajectories, which are primarily used for the 3D printing of polymer-based composite materials. The use of advanced printing methods will introduce a new dimension of uncertainty, resulting in an entirely new set of challenges pertaining to the occurrence of printing imperfections that must be considered and resolved [50,51].

### 2.3. Simulation Prior to Printing

Today, it is necessary to eliminate the waste of limited global material resources and maximize their potential. The advanced simulation tools follow the methodology of Wolfs et. al. [37] and have been in use since 2010’s 3DCP technology. 

To evaluate a pre-printed structure, commonly used numerical software is used which is based on the finite-element-method (FEM), such as Abaqus or Ansys [52]. To simulate the flow behavior during the extrusion and deposition process, computational-fluid-dynamics (CFD) is used [53]. It is important to note that simulation has its limitations, and it uses idealized and simplified properties of the material and printing process. The calculation’s numerical analysis disregards more complex material properties, such as thixotropic behavior, and is restricted to the linear region. Moreover, process parameters such as pumpability and extrudability are not addressed. However, it is an efficient tool that can save approximately 60% of the material, 30% of additional costs, and unaccountable time of human resources. Grasshopper (GH), a graphical algorithm editor with Voxel-Print or Cobra-Print extensions that comes as a plug-in for Rhinoceros surface modeller, is the primary tool for simulating the printing process. The Voxel-Print handles three general inputs, namely material properties, print settings, and geometry parameters and adheres to the general methodology proposed by Wolfs et. al. [27,54]. 

The methodology is comprised of experimental results and the numerical evaluation of the mechanical behaviour of early age concrete. Based on experiment evaluation, the Mohr—Coulomb theory has been applied and extended with time depending on material properties’ development. The output from the Voxel-Print plugin is an FE input file that retranslates the above-mentioned into Abaqus software [37]. The Cobra-Print plugin in Grasshopper extends the creation of the existing numerical modelling input file in Abaqus (known from the Voxel Print plugin) with advanced options such as Cobra Slicer and basic settings (including element dimensions, cross-sectional, and segment setup), extended settings (including path, bead width, roundness, interaction type, element type, and processor setup), mesh check, and print speed modification [52].

### 2.4. Novel Contribution of This Study

While 3D printing of concrete has great potential, there are still significant obstacles to achieving great buildability and high-quality prints. Large objects may collapse prematurely during solidification, which is one of the major limitations. In contrast to the works presented in the literature review, this study focuses on the 3D printing of material containing a large aggregate fraction. This kind of material has the potential to increase the strength of the layers and thus demonstrate the potential of printable concrete. By analyzing the material’s characteristics and adjusting the printing parameters, the success of the print can be determined. This study identifies appropriate input parameters for the aggregate material, thereby reducing the likelihood of printing failures. These findings can serve as a valuable guide for 3D concrete printing using aggregate materials, and similar simulations can be used to evaluate the expected behaviour of other materials. 

## 3. Methods of Printing Design

Before performing a real 3D print of concrete, it is essential to determine the appropriate printing parameters, as discussed previously. This study will evaluate the influencing factors on print success, attention is paid to the material with aggregates. The same procedure can be applied for materials of a different composition if specific analyses are not available.

### 3.1. Identification of Influencing Factors and Their Scaling

The output of the 3D concrete printing process is the printed structure’s height, which is a fundamental indicator evaluating the quality of the performed print. The process is influenced directly by uncontrollable and controllable influencing factors.

**Material properties (uncontrollable influencing factor)**

Uncontrollable factors can be traced but not directly influenced by print settings. In most instances, these factors relate to the characteristics of the printed material. To successfully perform a simulation that matches the actual material properties, the behavior of these factors must be precisely defined mathematically. Typically, these factors depend on the age of the material, which in our case is concrete. To simplify the simulation and reduce the required Hardware (HW), constant values were assigned to cohesion, density, friction angle, dilation angle, and Poisson’s ratio. Thus, only the Young’s modulus factor is described by a time-dependent function (t). Table 3 contains the formulations of the material properties based on the authors’ prior work and review.

- Young’s modulus of elasticity was determined by calculating the measured values σ_n_ and Ɛ at various times ranging from 20 to 45 min after wet mixing at 5 min intervals. For each time, three values were obtained, and the average value was calculated. A simple linear regression was used to determine the time evolution of Young’s modulus, where the independent variable is the time course, and the dependent variable is Young’s modulus [7].
- The cohesion behavior is time-dependent, the same for Young´s modulus, for material with coarse aggregate, which was obtained by evaluation of Shear box test. Cylindrical specimens were tested at the appropriate time intervals: from 20 min after wet mixing to 120 min at approximately 20-min intervals, with a horizontal displacement of 12 mm and nominal normal loads 5, 15, and 25 N plus the dead weight of a part of the specimen above the shear zone. The mixture with a coarse aggregate exhibited bilinear behavior, which would be difficult to implement in simulations. Therefore, the cohesion value was chosen as a constant based on the mean of all measurement values resulting in c=0.00429 [7]. Nota bene: the authors of study [24] recommended modelling cohesion behavior with constant and default settings.
- The internal friction angle of a material is related to cohesion due to the Mohr—Coulomb failure criterion, where the friction angle indicates the slope of the cohesion course; therefore, the average value is derived from measured values in previous authors’ studies [7], where the value is significantly higher for aggregate material compared to soft grained material.
- The material’s plastic behaviour can be analysed using the dilatancy angle; however, in 3DCP technology, the occurrence of plastic deformation typically results in print failure. Consequently, it is of little significance in terms of 3D printing, and its average value, based on a review of literature especially in study [31] where this phenomenon was discussed, was adopted.
- The Poisson’s ratio indicates the proportional transformation of a specimen under load, where the value of Poisson´s constant for fresh concrete was determined to be 0.3. Based on the literature review and the behaviour of Mix 1, the value was kept unchanged.

**Process parameters (controllable influencing factors)**

Controllable influencing factors can be set and thus influence the printing; therefore, these factors can be used to optimize the printing process. The purpose of this study is to determine the optimal setting of these factors for analysed material, Mix 1. A total of three controllable factors were identified and a range of possible settings, see Table 4.

Controllable influencing factors are continuous quantities that can take any value within the interval specified (column “Scale” in Table 4). Different combinations of setting these values will produce distinct print properties and the simulation can be performed for a limited number of combinations. The selection of such combinations for simulations is therefore crucial for the quality of the results of the study, which adequately characterizes the behaviour of the entire design space (Section 3.2).

**Print geometry (controllable influencing factor)**

**Figure 2:**
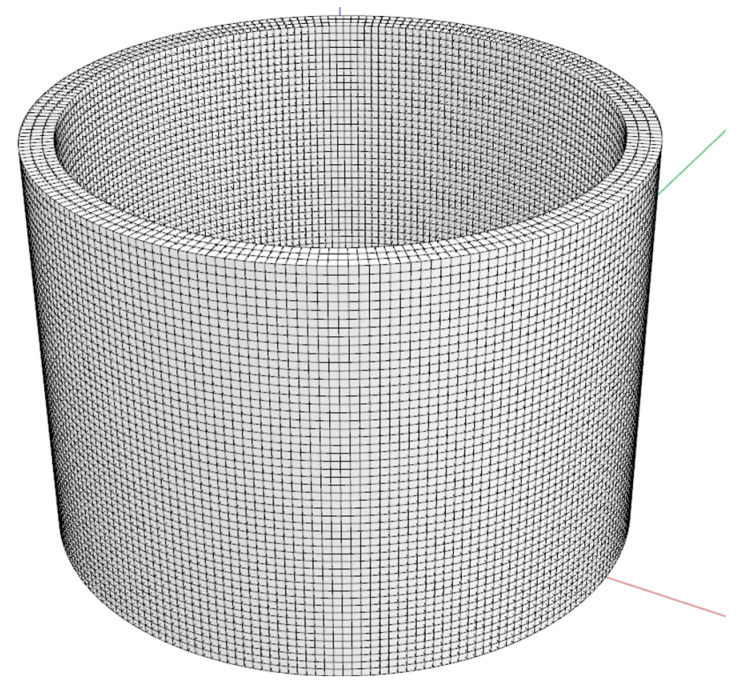
Hollow cylinder geometry with dimensions of 500 mm height and 700 mm in centreline diameter.

As stated in Section 2.1, to investigate and evaluate pure buildability the hollow cylindrical print geometry (Figure 2) is the most suitable in comparison with other geometry bodies. A further benefit of the selected print body is the elimination of the so-called minimum print radius. Moreover, a minimum print radius integrated into the print geometry can cause local plastic cracks that can lead to global print failure [55]. The characteristics of print geometry were specified in greater detail (Table 5) based on findings from a literature review (Table 2) and material properties (Table 1) [7]. The geometric characteristic is enriched by a parameter called perimetric complexity. Originally, perimetric complexity was used to measure the complexity of binary images [56]. It is defined as the sum of the inner and outer perimeters of the foreground surface divided by 4π. This parameter is a metric in numerous shape analysis applications such as the human letter identification, handwriting recognition, etc. [57,58,59]. It can be then associated with the cross-sectional area of the 3D printed structure and used to describe the geometry of the layers of the printed solid. Consequently, the application of machine vision and machine learning to 3DCP technology for the evaluation of printed geometry is outlined.

### 3.2. Selection of Combinations for Performing the Simulation

**Figure 3:**
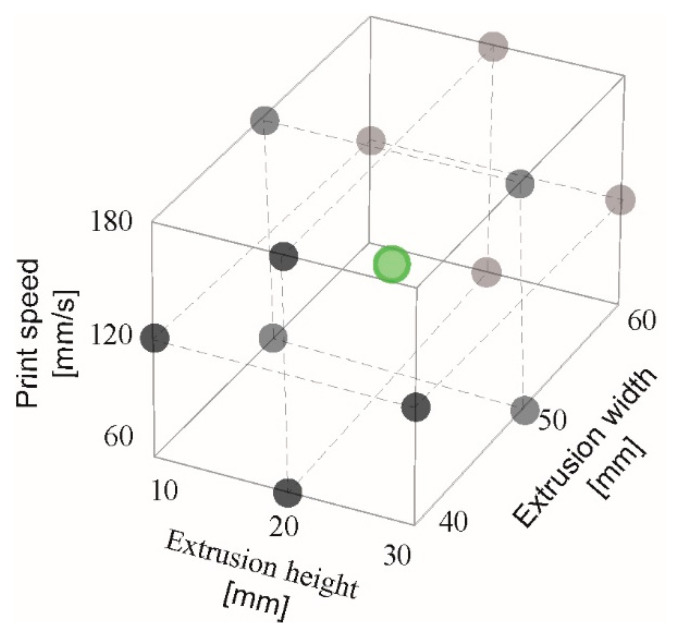
Simulation points layout for three factors—Box—Behnken cube design.

Combinations of controllable influencing factors for simulations are determined using the theory of Design of Experiment (DOE) principles implemented in Minitab software [60]. It can be assumed that the DOE model with factors specified in Table 4 will also include nonlinear dependencies, so a second-order model is considered. DOE is therefore implemented using the Box—Behnken design, which is appropriate for models with at least three variables [61]. The model includes central points that can provide information regarding curvature. The central points can be considered because the factors are quantitative. Therefore, it is possible to determine the middle level of each factor and the middle of the whole system. Because the experiment is carried out via simulation, the measurement error has no effect on the findings. Therefore, only one replication is performed; multiple replications would bring in identical results. Using the Box—Behnken method for three factors, an experiment (in this case a simulation) for 13 combinations of controllable factors was proposed. Figure 3 depicts the coverage of the tested region with three factors, with the center point highlighted in red. 

### 3.3. Simulation Conditions

This study simulates the 3DCP process for a material derived from previous research [15], which contains coarse aggregate with a maximum particle size of 8 mm under ideal conditions for both the material and print processes. In consideration of computational cost, the number of segments per layer, and the mesh configuration resulting in collapse height, the optimal settings for simulation inputs are discussed in light of the study [52]. In accordance with the defined rules, the suitable configuration for a mesh element was determined to be 3 elements in width $n_{1}$, 2 elements in height $l^{e}$ and 10 mm element length $l^{e}$. Considering the objective of this study, which involves the use of the CC method that produces a rectangular cross section of the print trace, the mesh element type was set to C3D8, an 8-node linear brick (includes the defined above-described element size) to construct the FE mesh, and the bead width factor was set to 0. Due to the oversimplification of a physical printing (temporary asymmetric loading conditions) with a low number of segments per layer, it is important to select an appropriate number of segments per layer. For a more precise estimation of the failure height, using more than the S1 segment is recommended. For the needs of this study, the S8 was chosen for its good balance with the optimal setting of the simulation mentioned previously. In addition, the absence of automatic stabilization mechanism results in more reliable failure height measurements for the 3DCP process. The interaction type of a layer was set to constant-based to include the material cohesive behavior. All input settings were preserved in Table 6. The simulation disregards aspects that can negatively impact the printing process, e.g., the translation of the nozzle to the next layer, which contributes to an increase in local vertical stress and can contribute to plastic failure or elastic buckling. Horizontal deviation of the deposited material from the previous print path can result in a premature critical buckling load [27,62].

## 4. Results and Discussion

Table 7 shows the individual combinations as well as the simulation results. The experiment (resp. simulation) was performed for 13 different settings of three input parameters, and the influence of these parameters on printing success was assessed.

As mentioned above, the print was assessed for different settings of the three input factors (v, $E_{w}$ and $E_{h}$). The criterion for printing success is the height of the printed object (H), and the goal is to reach the highest possible height of the object. The indicated print height (H) corresponds to the situation before its final collapse. It should be noted that material subsidence was also taken into account for the assessment. The best result (H=350.549 mm) was obtained for the combination of input factors No.10, this is the middle setting of v, maximal $E_{w}$ and minimal $E_{h}$.

**Figure 4:**
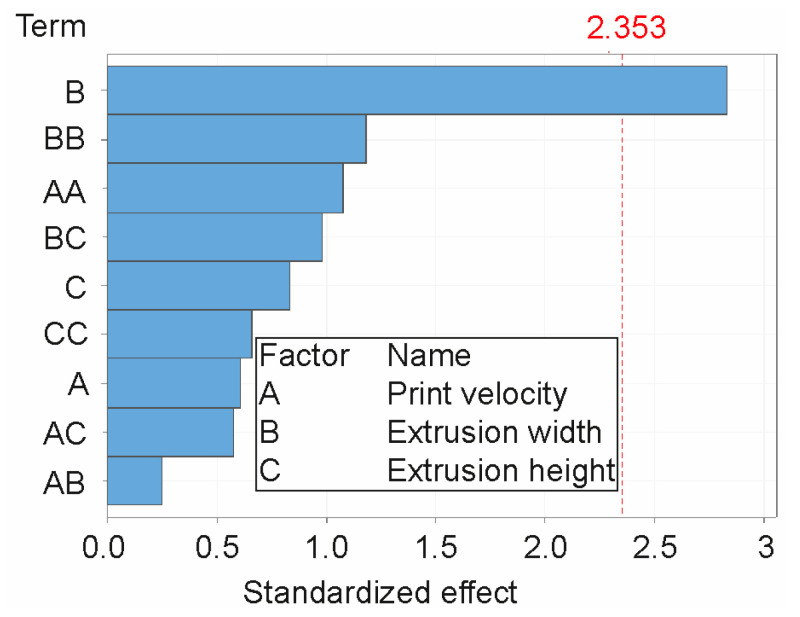
Pareto chart of the standardized effects of factors A, B, C where the labelled reference level crossed only parameter B (response is print height H in significance level α=0.1).

Using regression analysis, the influence of individual factors on print height was evaluated. The R^2^ value indicates that the model explains 83.04% of variability in the data. Table 8 provides a summary of the fundamental characteristics of the regression model. For the regression model, individual parameters and their combinations were considered. The interpretation of the individual values can be found in the software documentation or in the standard documents pertaining to regression analysis. The null hypothesis that the coefficient of an individual factor is equal to zero was tested. Rejecting the null hypothesis indicates the existence of a response between the factor (v, $E_{w}$, $E_{h}$ or their combination) and the print height H. The P-value is crucial to assess the influence of factors. $E_{w}$ is the only significant factor at a significance level of α=0.1 (0.066 < 0.1) based on the results. According to the tests, other factors and their combinations are statistically insignificant; see Table 8. Figure 4 (the Pareto chart) displays the influence of the factors using the standardized effect, which is the absolute value of the T-value used to test the null hypothesis that effect is zero (Figure 4). There are significant factors whose bars cross the reference line with the value 2.353 at the significance level α=0.1.

**Figure 5:**
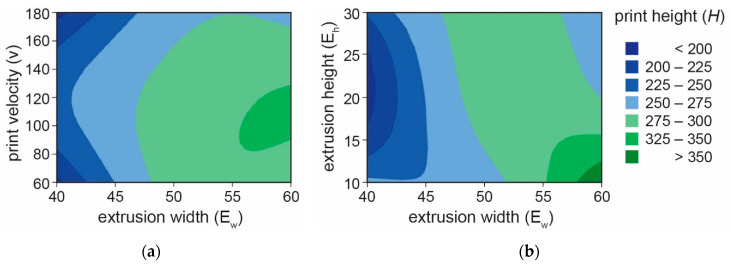
Contour plot of maximum print height dependency on combinations of extrusion width $\left(E_{w}\right)$ and velocity (v) (**a**), and combination of print width ${(E}_{w})$ and height ${(E}_{h})$ (**b**).

The results are depicted in contour plots (Figure 5), where the only significant factor is B (extrusion width $E_{w}$) in conjunction with combinations of the remaining factors (AB—print velocity v with Extrusion width $E_{w}$, BC—extrusion width $E_{w}$ with extrusion height $E_{w}$).

Figure 5b depicts the significant level of print height (H) correlated with the factor combination, with the highest point occurring when factor B = 60 mm (extrusion width $E_{w}$) and the value of factor C = 10 mm (print velocity v) are set to their respective values. While the less significant combination of factors AB (extrusion width $E_{w}$ and print velocity v) with values approximately B = 55–60 mm and A = 90–120 mm is illustrated in Figure 5a.

**Figure 6:**
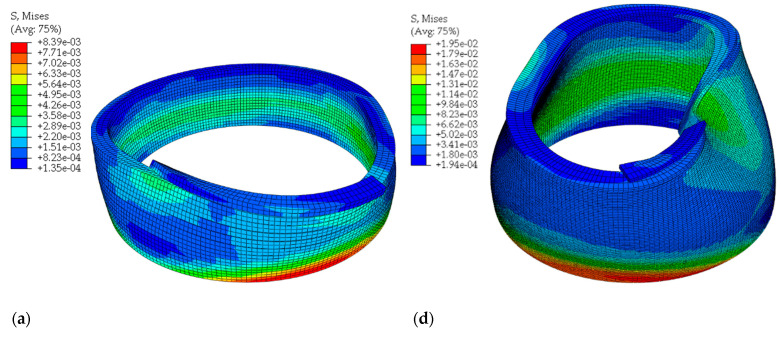

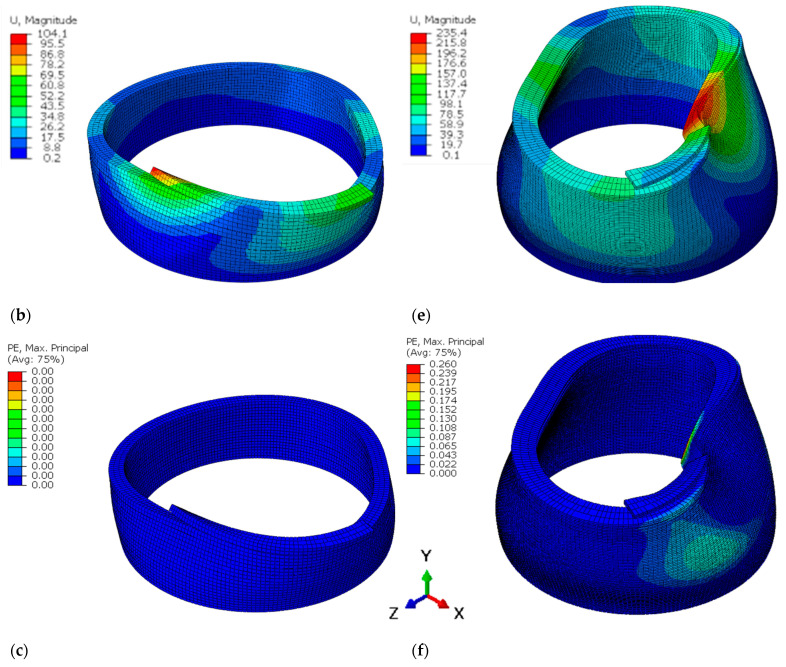
Numerical results showing the asymmetric failure of a cylindrical geometry for two extremes: minimum (**a**–**c**) and maximum (**d**–**f**) buildability with stress, deformation, and plastic deformation visualization. The results are highlighted in Table 7 (maximum green and minimum red).

Numerical models resulted in asymmetric deformation (Figure 6b,e) during the failure occurrence, where the print height difference was approximately ∆H=160 mm. For the combination of influenced factors, No. 2, the clear elastic buckling failure, occurred according to the deposition of the 11th layer (Figure 6c), while for No. 10 the combination of elastic and plastic collapse occurred when the 39th layer was deposited. The pressure of upper layers (Figure 6d) caused the plastic yielding, firstly occurring in the 12th layer, which initiated the elastic collapse in the buckling failure mode (Figure 5f). The parameter of print velocity with combinations of larger print can significantly affect the buildability of a printed structure in correlation with the hardening development of a material. This specific material from the study [7] is intended for large-scale printing. In that case, the material behaviour would be significantly different.

## 5. Conclusions

In the context of 3D construction/concrete printing, this study examined the theoretical limits of a mixture with coarse aggregates up to 8 mm. In particular, the impact of the identified influencing factors, their combinations, and their scaling on the 3D printing process in a virtual environment were investigated. Uniquely, all possible combinations (design space) of the selected influencing factors (printing speed, extrusion width, and extrusion height) were investigated systematically using the Design of Experiment approach, specifically the Box—Behnken method, resulting in a reduction in the number of simulations to 13. The study improves the efficiency of the 3D printing process of this material through numerical simulations, the results of which were used to predict the behavior of the compound during printing. The study resulted in, among other things, the targeted setting of controllable parameter values that, in theory, increase buildability.

Specific findings were as follows.

- The technology of 3D concrete printing is currently facing multidimensional challenges that need to be overcome. From a practical standpoint, the trial-and-error approach is a process that requires a great deal of energy, materials, time, and human resources. It is also very costly for numerical simulations, an implementation on the order of hours/days for the CPU, depending on the complexity of the printed object. Therefore, it is important to reduce the number of simulations required to explore the entire design space using a DOE (Design of Experiment) approach.
- Extrusion width is a significant factor; other factors and their combinations are statistically insignificant according to the tests. The input combinations used for the simulation resulted in a theoretically correct combination of process parameters and were based on the standardized effect at significance level α = 0.1 (0.066 < 0.1).
- A non-monotonic relationship was found for the parameters of the printing process, namely layer height, layer width, and printing speed.
- The prediction of buildability can thus be considered a non-trivial problem.

**General Findings**

- Most of the studies did not provide a complete material model that could be the new established standard in the 3DCP technology research community. This would avoid an unnecessary waste of human resources and raw materials in the case of follow-up research.
- It would be useful to include other parameters in geometry and material to the DoE, but this leads to a huge number of simulations, which is not realistic. The goal is to be able to control all parameters, where it seems best to use machine learning.

**Future Work**

- The behavior of coarse aggregate in the fresh state needs to be further investigated. The sensitivity of buildability needs to be studied in terms of the natural (irreducible) variability of material parameters (up to 20% natural variability in concrete) to avoid the occurrence of imperfections due to the scale and complexity of the print geometry. In addition, controllable parameters that can compensate for the above irreducible variability should be considered to ensure exposure even under the existing uncertainty conditions. In particular, bio-inspired geometries, generative lattice structures, topologically optimised structures, etc., fulfil these sub-goals.
- In the future, printing shape-complex geometries will require a different approach to the strategy and methods used to generate print trajectories. Currently, they are widely used, especially in the 3D printing of polymer-based materials. The use of advanced printing methods will introduce a new dimension of uncertainty, leading to entirely new challenges to consider and solve. This is where the development of machine vision and machine learning lends itself to the evaluation of print geometry, where the parameter of perimetric complexity can be combined with the cross-sectional area of the 3D printed structure and used to describe the geometry of the layers of the printed body.
- Based on the above, it will be possible to create a robust formulation of a digital twin for the 3D concrete/structure printing process and the associated digital concrete model.

The next step in this research will be to test and refine these conclusions using complex geometries. In doing so, the material model will be extended to include more parameters that can be influenced, and the number of simulations will take into account more time-dependent variables where additional correlations can be expected.

## Figures and Tables

**Table 1:** Material characteristics of cement-based materials for 3DCP—literature review.

No.	$\rho \left(t\right)\;\left[kg/m^{3}\right]$	$v\left(t\right)$ [-]	φ[°]	ψ[°]	E(t) [MPa]	c(t) [MPa]
[38]	2255	0.3	34.5	13	0.001578t+0.112260	0.0000780t+0.005790
[31]	2100	0.3	1, 6, 7	12, 20	0.001705t+0.039	0.00006t+0.026
[27] Mix 1	2000	0.24	20	13	0.0120t+0.020	0.0024t+0.004
[27] Mix 2	2000	0.24	20	13	0.0240t+0.040	0.0006t+0.001
[27] Mix 3	2000	0.24	20	13	1.2000t+0.080	0.0018t+0.005
[27] Mix 4	2000	0.24	20	13	0.9600t+0.060	0.0120t+0.020
[27] Mix 5	2000	0.24	20	13	0.8400t+0.040	0.0600t+0.010
[27] Mix 6	2000	0.24	20	13	0.6000t+0.020	0.2400t+0.040
[30]	2100	0.3	−	−	0.0486t+0.0026	−
[37]	2070	0.24	20	13	0.0012t+0.078	0.0000508t+0.003
[28]	2100	−	−	−	0.0026t+0.048	−
[27]	2500	0.22	20	13	0.0032t+0.048	0.00003t+0.004
[7] Mix 1 *	2218	−	54.72	−	0.0013t+0.0365	−
[7] Mix 2 *	2130	−	55.4	−	0.0094t+0.2564	−

* Previous work of the authors.

**Table 2:** Printing parameters of cement-based materials for 3DCP—literature review.

No.	t [min]	v [mm/s]	Ht [mm]	Wt [mm]	Nozzle Type: Circular (Ø), Rectangular (a × b) [mm]	Print Geometry			
						Hollow Cylinder (Centreline Radius) [mm]	Square (a × b)/Wall (l) [mm]	Cone (Centreline Radius 1,2) [mm]	Dome (Radius) [mm]
[38]	0–45	60	10	40	25	12; 5	−	R1 = 100, 75, 50 R2 = 200, 150, 100	200, 150, 100
[37]	−	83.33	10	40	−	250	−	−	−
[27] Mix 1	−	80	12	25	−	100 (with inclination 12°)	−	−	−
[27] Mix 2	−	80	12	25					
[27] Mix 3	−	180	10	40					
[27] Mix 4	−	180	10	40					
[27] Mix 5	−	180	10	40					
[27] Mix 6	−	180	10	40					
[30,42,43]	5–65	104.16	9.5	55	Ø = 6–25, 30 × 10, 40 × 10	−	250 × 250, 500 × 500, 625 × 250 l = 800	−	−
[28]	−	~ 60	10	40–55	−	250	250 × 250	−	−
[18] *	−	80	10	35–60	−	−	250 × 250, 500 × 500	−	−
[40]	−	60	10	30	−	250	−	−	−
[44]	−	84.5–104	10	40–55	−	250	250 × 250	−	−

* Geopolymer mortar.

**Table 3:** Setting material properties.

Influencing Factor	Mix 1	Remark
Young’s modulus [MPa]	$E\left(t\right)=0.0013t+0.0365$	UUCT [7]
Cohesion [MPa]	0.00429	Shear box test [7] *
Density [kg/m^3^]	2218	Volumetric method
Friction angle [°]	54.72	Direct shear test [7]
Dilation angle [°]	13	*
Poisson constant [-]	0.3	*

* Constant values and recommendations obtained from review [24,31].

**Table 4:** Setting controllable influencing factors.

Influencing Factor	Scale	Remark
Print speed (v)	60–180	mm/s
Extrusion width ($E_{w}$)	40–60	mm
Extrusion height ($E_{h}$)	10–30	mm

**Table 5:** Setting print geometry properties.

Geometry Parameter	Hollow Cylinder
Geometry height [mm]	500
Maximum overhang [°]	0
Perimeter length [mm]	2199
Perimeter length variability [-]	COV * = 0
Perimetric complexity [-]	1 **
Geometry volume [m^3^]	0.0440/0.0659
Bounding box ratio [-]	0.66
Minimal horizontal radius [mm]	R > 0 *** R = 0 ****

* Coefficient of variation, ** Circular geometry, *** Extrusion based method, **** CC method.

**Table 6:** Cobra-Print parameters set as a constant for each simulation.

Input Settings		Remark
Element length [mm]	10	$l^{e}$
Elements in width	3	$n_{1}$
Elements in height	2	$n_{2}$
Number of segments	8	S8
Extended settings		
Bead width factor	0	-
Interaction type	Contact-based	-
Element type	8-node linear brick	C3D8
Processor CPUs	8	-
Numerical stabilization	OFF	-

**Table 7:** Results of the simulations.

No.	v [mm/s]	$E_{w}$ [mm]	$E_{h}\mathrm{[mm]}$	H [mm]
1	60	40	20	191.043
2	180	40	20	189.835
3	60	60	20	283.219
4	180	60	20	264.265
5	60	50	10	285.645
6	180	50	10	244.727
7	60	50	30	285.557
8	180	50	30	285.506
9	120	40	10	257.226
10	120	60	10	350.549
11	120	40	30	229.959
12	120	60	30	253.671
13 *	120	50	20	285.032

* Central point.

**Table 8:** Coefficients for describing the regression model.

Term	Coef	SE Coef	T−Value	*p*-Value	VIF
Constant	285.0	35.4	8.05	0.004	-
v	−7.6	12.5	−0.61	0.585	1.00
$E_{w}$	35.5	12.5	2.83	0.066	1.00
$E_{h}$	−10.4	12.5	−0.83	0.466	1.00
v∗v	−25.2	23.4	−1.08	0.360	1.35
$E_{w}*E_{w}$	−27.7	23.4	−1.18	0.322	1.35
$E_{h}*E_{h}$	15.5	23.4	0.66	0.554	1.35
${v*E}_{w}$	−4.4	17.7	−0.25	0.818	1.00
${v*E}_{h}$	10.2	17.7	0.58	0.604	1.00
$E_{w}*E_{h}$	−17.4	17.7	−0.98	0.398	1.00

## Data Availability

The data presented in this study are available on request from the corresponding author.
